# Possible mechanisms of pollination failure in hybrid carrot seed and implications for industry in a changing climate

**DOI:** 10.1371/journal.pone.0180215

**Published:** 2017-06-30

**Authors:** Melissa Ann Broussard, Flore Mas, Brad Howlett, David Pattemore, Jason M. Tylianakis

**Affiliations:** 1 School of Biological Sciences, University of Canterbury, Christchurch, New Zealand; 2 The New Zealand Institute for Plant & Food Research, Christchurch Mail Centre, Christchurch, New Zealand; 3 The New Zealand Institute for Plant & Food Research, Waikato Mail Centre, Hamilton, New Zealand; 4 Department of Life Sciences, Imperial College London, Silwood Park Campus, Ascot, Berkshire, United Kingdom; Institute of Botany, CHINA

## Abstract

Approximately one-third of our food globally comes from insect-pollinated crops. The dependence on pollinators has been linked to yield instability, which could potentially become worse in a changing climate. Insect-pollinated crops produced via hybrid breeding (20% of fruit and vegetable production globally) are especially at risk as they are even more reliant on pollinators than open-pollinated plants. We already observe a wide range of fruit and seed yields between different cultivars of the same crop species, and it is unknown how existing variation will be affected in a changing climate. In this study, we examined how three hybrid carrot varieties with differential performance in the field responded to three temperature regimes (cooler than the historical average, average, and warmer that the historical average). We tested how temperature affected the plants' ability to set seed (seed set, pollen viability) as well as attract pollinators (nectar composition, floral volatiles). We found that there were significant intrinsic differences in nectar phenolics, pollen viability, and seed set between the carrot varieties, and that higher temperatures did not exaggerate those differences. However, elevated temperature did negatively affect several characteristics relating to the attraction and reward of pollinators (lower volatile production and higher nectar sugar concentration) across all varieties, which may decrease the attractiveness of this already pollinator-limited crop. Given existing predictions of lower pollinator populations in a warmer climate, reduced attractiveness would add yet another challenge to future food production.

## Introduction

Insect-pollinated crops comprise approximately one-third of the global food supply [1]. Many of these plants owe their present uniformity [2], disease resistance [2], and high yields [3–6] to hybrid production systems, including carrot, tomato, onion, melons, squash, brassicas, and eggplant [7]—together totaling nearly 20% of global crop production [8]. Because these production systems rely on crossing two parent lines, one of which is rendered male-sterile by hand-emasculation or genetic techniques, they are even more reliant on insect pollinators than their open-pollinated counterparts, do not require insects to cross from one parent line to the other [9–12]. Global reports of declines in many pollinator communities [13; 14], changing climate shifting pollinating insects' active time away from peak bloom [15], and that pollinator reliance has been linked with reduced yield stability [16], indicate that hybrid systems may be at greater risk from additional disturbances than open-pollinated systems.

Yields of any given hybrid crop can vary significantly between varieties and also from year to year. In itself, this generates economic uncertainty, but it also makes determining which cultivars are best suited to the changing environment a difficult task. When hybrid crops that are grown for seed fail to produce adequate yields, pollinators are often blamed [10], particularly as male-sterile plants, which have no pollen reward, are notoriously unattractive to honey bees [9; 12; 17]. However, it is unclear if the observed poor yields are due to the lack of pollen, other characteristics that have inadvertently been selected for during the process of crop breeding and selection, or a combination of plant traits and environmental variables.

Traits that affect the yield of insect-pollinated plants can broadly be placed into two categories: plant fertility and plant attractiveness. Characters surrounding plant fertility are intrinsic to the plant, such that increased pollinator activity will not improve yield. For example, if flowering of the male-fertile and male-sterile lines is poorly synchronized, yields can suffer as the cross-pollination window may not overlap sufficiently. In such cases, cultural measures to synchronize the lines even by a few days can substantially increase yield [18]. Pollen viability has been a recurrent problem in hybrid crops as well, with inbreeding depression often causing pollen viability to drop to 50% or less before it ever leaves the flower [19–21]. This initial difficulty can be further exacerbated by heat and water stress during critical periods of plant development, which can further degrade pollen fertility [20; 22]. Female receptivity is also important, as flowers that are too young, too old, or damaged by suboptimal temperatures will set little seed even if pollen viability is high [23].

Characters surrounding plant attractiveness, in contrast, are those which influence pollinator visitation. The quality of the nectar reward is foremost for ensuring return visits [24–26]. Honey bees prefer nectar rewards between 30 and 50% sugar w/w [25], but lower concentration sources at higher volumes per flower may be chosen over many low-volume, high-concentration sources [25]. Nectar production is altered in high-temperature conditions, typically resulting in lower volumes and higher concentrations [25], but hot conditions can also alter the production of secondary compounds, such as phenolics [27], which can result in a very different flavor palette to potential insect visitors. Volatile compounds emitted by the plant are also important, as they play a key role in attracting pollinators to the flower initially [25]. Although the volatiles comprising the floral bouquet of numerous plant species have been cataloged, little is known about how these compounds are perceived by pollinators, and less still is known about how they respond to changes in temperature, individually or in aggregate.

In order to achieve successful pollination, a plant must be both fertile (able to receive pollen and set seed), and attractive to pollinators. To understand how climate change may affect pollination, we must therefore look at factors relating to both overarching categories. To address these broad questions, we chose to focus on hybrid carrot production because, despite being a generalist flower pollinated by hundreds of insect species [28–31], hybrid carrot is known for its low seed set [32] and lack of pollinator attractiveness [10; 33]. In addition, seed production for carrot occurs in areas not optimal for carrot growth in order to avoid genetic contamination from wild carrot [34; 35], which can readily cross into cultivated varieties and reduce agronomic quality of progeny [34; 35]. As a number of major carrot seed producing regions are located in temperate, semi-arid areas [36–38], additional temperature variability may negatively affect seed production. The combination of environmental stress and poor pollination in present-day hybrid carrot make it a promising model for future conditions experienced by hybrid crops, and examining the mechanisms of current pollination failure may highlight future vulnerabilities both carrot and hybrid crops in general.

The objectives of this study were to 1) test the effect of temperature on temporal patterns of plant traits that might predict performance in the field, including bloom phenology, seed set, pollen viability, nectar quality (both sugars and phenolic compounds), and floral volatiles and 2) examine if these effects differ across varieties with a range of historical yields to test whether varietal differences are reduced or exaggerated by warming, and 3) use this information to determine which factors are important in present-day pollination failure, and which may be important given a changing climate.

## Methods

This study was conducted in New Zealand as it is one of the world's largest producers of carrot seed [39]. We exposed carrot (*Daucus carota* L) plants to experimental temperature treatments, and measured characteristics relating to their innate ability to produce seed ('Plant Fertility Metrics', below) as well as several metrics that may affect their attractiveness to pollinators in the field ('Plant Attractiveness Metrics', below). To assess the extent to which these characters contribute to differences in yield and how they may respond to climate warming, we examined the correlations between each one and plant variety, temperature, and time-of-day in generalized linear mixed-effects models (GLMMs), generalized additive mixed-effects models (GAMMs) or ordination-based tests.

### Plant material

In order to determine how floral receptivity and pollen viability vary with time-of-day (a known source of variation in floral traits [25; 40]) and temperature, we grew the male-fertile and cytoplasmically male-sterile (brown anther type) parents of three lines of Nantes-type hybrid carrot for hand-pollination trials and measures of pollen viability. These three lines had previously been observed to perform poorly (172 ± 43 kg/ha), average (377 ± 17 kg/ha), and well (607 ± 87 kg/ha) in the field (hereafter referred to as 'poor', 'average' and 'excellent' lines); we chose this gradient to attempt to tease out the cause(s) of the differential performance in the field, and to have a range of yields to assess the effects of temperature. Seeds for each line were sown in trays in February 2015, during the southern hemisphere summer. When plants had germinated, we transplanted them individually into 3L pots filled with potting mix and slow-release fertilizer (Canterbury Landscape Supplies). Each line had 100 male-sterile plants and 75 male-fertile plants potted out, and these were kept outdoors in ambient conditions until flowering began.

In order to minimize the effect of temperature on plant physiological processes other than flowering, we moved plants to temperature treatments after the umbels had formed, and just prior to flower opening. The three temperature treatments simulated cool, average, and warm seasons via shade houses, unheated glasshouses and heated glasshouses, respectively. Two separate buildings were used for each temperature treatment, and the plants were equally divided between them. To accurately record conditions experienced by the carrot flowers, we placed temperature and relative humidity probes (onset HOBO Prov2 temp/RH meters) at chest height in each of the six locations. Temperatures were extracted from the data loggers and recorded as temperature at the sample time (S2 Fig), average temperature during the 24-hour period prior to the sample time, and average temperature during each plant's time in the glasshouse prior to sampling. Models were run with each measure of temperature sequentially, but in all cases, the 24-hour period had the highest predictive value, so only this measure was used.

Every day, we checked plants for floral stage, and, when the petals had turned white and the outer whorl of flowers had just begun to open, they were randomly assigned to a temperature treatment. Male-sterile plants were also assigned to one of seven time treatments (4am, 8am, 11am, 2pm, 5pm, 8pm, and 11pm) for hand-pollination. Selection of treatments was done without replacement, so that male-sterile plants were always equally distributed between the three temperature treatments and each time-temperature combination received the first replicate before proceeding to the second, third, and fourth (252 male-sterile plants total). Male-fertile plants were preferentially assigned to locations where male-sterile plants required pollination.

Once selected, we bagged the primary umbel of each male-sterile plant with 1mm mesh to prevent insect pollination and, as an extra precaution, placed plants into a 1.5m^3^ fine mesh cage in each of the six locations. Male-fertile plants were left unbagged inside the cage. As a further precaution, we placed yellow sticky cards in each cage to trap any flying insects that entered.

### Plant Fertility Metrics

#### Phenology

In order to properly hybridize in the field, both the male-sterile and male-fertile lines must bloom simultaneously, and the male-fertile lines should, ideally, produce pollen for the duration of the male-sterile line's bloom time. To quantify this bloom synchrony, we checked the primary umbel of each potted carrot plant daily. Just prior to the opening of the outer whorl of umbellets, we recorded the date and assigned the plant to its treatment. We then calculated the number of days between seed sowing and flowering. Any plants that had not flowered after 365 days were recorded as having failed to bloom.

#### Seed set

To quantify changes in stigma receptivity across lines, temperature treatments, and at different times of day, we conducted a hand pollination experiment. Once pollen from male-fertile plants started to dehisce, hand-pollinations began. Every morning at 8am, we surveyed caged plants and any plant where the stigmas appeared receptive throughout the umbel was pollinated that day at its pre-selected time slot. To assist with visually identifying receptive stigmas, a photo guide was prepared the season prior by staining stigmas with alpha-napthyl acetate [41]. The primary umbel of each plant to be pollinated was unbagged and three umbellets were selected and tagged, one from each of the three whorls, as previous studies indicate that there may be differences in female fertility between the inner and outer umbellets [42]. As the 'medium' male-fertile line was the only one blooming throughout the sample period, we used pollen from this line for hand-pollinations of all male-sterile lines. For each time slot where a flower needed to be pollinated, we bulked together pollen from all the 'medium' male-fertile plants in the building. We took a subsample of this pollen, put it in a cryotube, immediately placed it in liquid nitrogen for later pollen viability assessment, and applied the remainder with a paintbrush to each stigma of each floret of the tagged umbellets of all flowers in the building needing pollination at that time. Plants were then rebagged and left in the glasshouse for a further 72 hours, to allow pollen tubes to reach the ovaries (typically 24–48 hours [10]), before being brought back outside to complete seed set.

Once the seed heads dried, we brought them back into the lab, and the seeds of the three tagged umbellets of each flower were counted. In addition, three untagged umbellets, one from each whorl, were examined as an unpollinated control for each plant.

#### Pollen viability

Subsamples of bulked pollen were stored in cryotubes in liquid nitrogen until the final pollination for each day (typically 11pm or 4am), when we transferred it to a -80°C freezer. When all pollen samples were collected, we transferred them to a second facility on dry ice and then immediately placed them in a second -80°C freezer until processing.

Pollen viability was assessed with fluorescein diacetate (FDA), which has previously been shown to have a strong correlation with *in-vivo* germination in carrot [43]. We thawed the cryotubes for 2–5 minutes and then washed the tube with 50μl FDA-sucrose solution (0.25% w/v FDA, 20% w/v sucrose) via a pipettor, with as much liquid as possible collected and slide mounted. We examined samples with a UV light microscope, counting 200 pollen grains across longitudinal transects of each slide. We categorized pollen as viable if it fluoresced bright green [44].

### Plant Attractiveness Metrics

#### Nectar quality

After volatile collection (if applicable, see below), but prior to pollination, we sampled each male-sterile plant for nectar. We followed the protocol described by Gaffney [31], dipping half of each umbel (~30 umbellets, with the umbel diameter being controlled for in analyses) into 40mL of distilled water twenty times, ensuring that the umbel was shaken off afterward to recover as much water as possible. We then immediately placed the dilute nectar in a freezer until further processing. To prepare the samples for high-performance liquid chromatography (HPLC), they were thawed, filtered to remove any large contaminants, and freeze-dried in 50mL falcon tubes. We then resuspended the samples in 1mL of methanol:water at a ratio of 1:1 and divided it in two parts: a 600 μL aliquot for nectar sugar analysis and a 400 μL aliquot for nectar phenolic analysis.

Sugar identifications were made via HPLC using a modified combination of the methods of Ruperez [45], Knudsen [46], and Knudsen and Li [47]. The 600μL aliquot was centrifuged at 14,000 rpm for 10 minutes. A 250 μl aliquot of the supernatant was placed directly into an HPLC vial. We then carried out the HPLC analysis using a refractive index (RI) detector (Waters^™^ Alliance 2690 HPLC with Waters^™^ 2414 RI detector). HPLC-RI analysis was carried out by injecting 10 μl of sample into an isocratic mobile phase of 70% acetonitrile in water with chromatographic separation (Econosphere^™^, Amino, 5μm, 4.6 x 250mm, Grace^™^) at 30°C and RI detection at 40°C. Unknown sugars were identified using retention times and response factors of known sugars (Sigma).

Phenolic composition of the nectar was also analyzed via combined liquid chromatography-mass spectrometry (LC-MS). The previously prepared 400μl aliquots were filtered with a Single Step^®^ vial 0.22 μm PVDF (Thompson^™^ Part No. 65531–200) filter. The LC-MS system consisted of a Thermo Electron Corporation (San Jose, CA, USA) Accela UHPLC pump, Thermo Accela Open Auto sampler (PAL HTC-xt with DLW), Finnigan Surveyor PDA plus detector and a ThermaSphere TS-130 column heater (Phenomenex, Torrance, CA, USA). Each of the 48 extracts was analyzed by two ion formation modes creating 96 data files, as follows. A 2μL aliquot of each prepared extract was separated with a mobile phase consisting of 0.1% formic acid in water (A) and 0.1% formic acid in acetonitrile (B) by reverse phase chromatography (Kinetex guard cartridge and Kinetex C18, 2.6 μ, 100 Å, 100 x 2.1 mm, Phenomenex, Torrance, CA, USA) maintained at 30°C with a flow rate of 200 μl/min. A gradient was applied: as 0–10 min/95%A, 13 min/60%A, 15-20min/5%A, 23-28min/95%A. The eluent was scanned by API-MS (LTQ, 2D linear ion-trap, Thermo-Finnigan, San Jose, CA, USA) with electrospray ionisation (ESI) in the negative mode. Data were acquired for precursor masses from m/z 120–1000 with up to MS3 product spectral tree formation. All data were processed with the aid of Xcalibur^®^2.20 (Thermo Electron Corporation) and an in-house Plant and Food Research database of chemical signatures.

#### Floral volatiles

We collected volatile organic compounds (VOCs) from the headspace of plants after the outer whorl of florets opened, but prior to pollination. Due to resource limitations, we could not collect volatiles from every plant, so a subsample was taken across the treatments. To achieve a good cross-section of the experimental treatments, we collected three separate datasets; one across varieties, one across temperatures, and one across times of day. For the variety dataset, we took 24-hour headspace collections for twelve plants (6 male-sterile, 6 male-fertile) from each of the three varieties in the average temperature treatment, totaling 36 samples. For the temperature dataset, we took 24-hour headspace collections from 6 'medium' male-sterile plants each in the cool and hot treatments, which were analyzed together with the 'medium' male-sterile samples in the previous dataset, totaling 18 samples (12 unique to this dataset). For the final dataset, in order to capture variation throughout the course of the day, we sampled six further 'medium' plants beginning at each of the seven time periods (3–5 hour headspace collection), for 42 total time-of-day samples.

**Fig 1:**
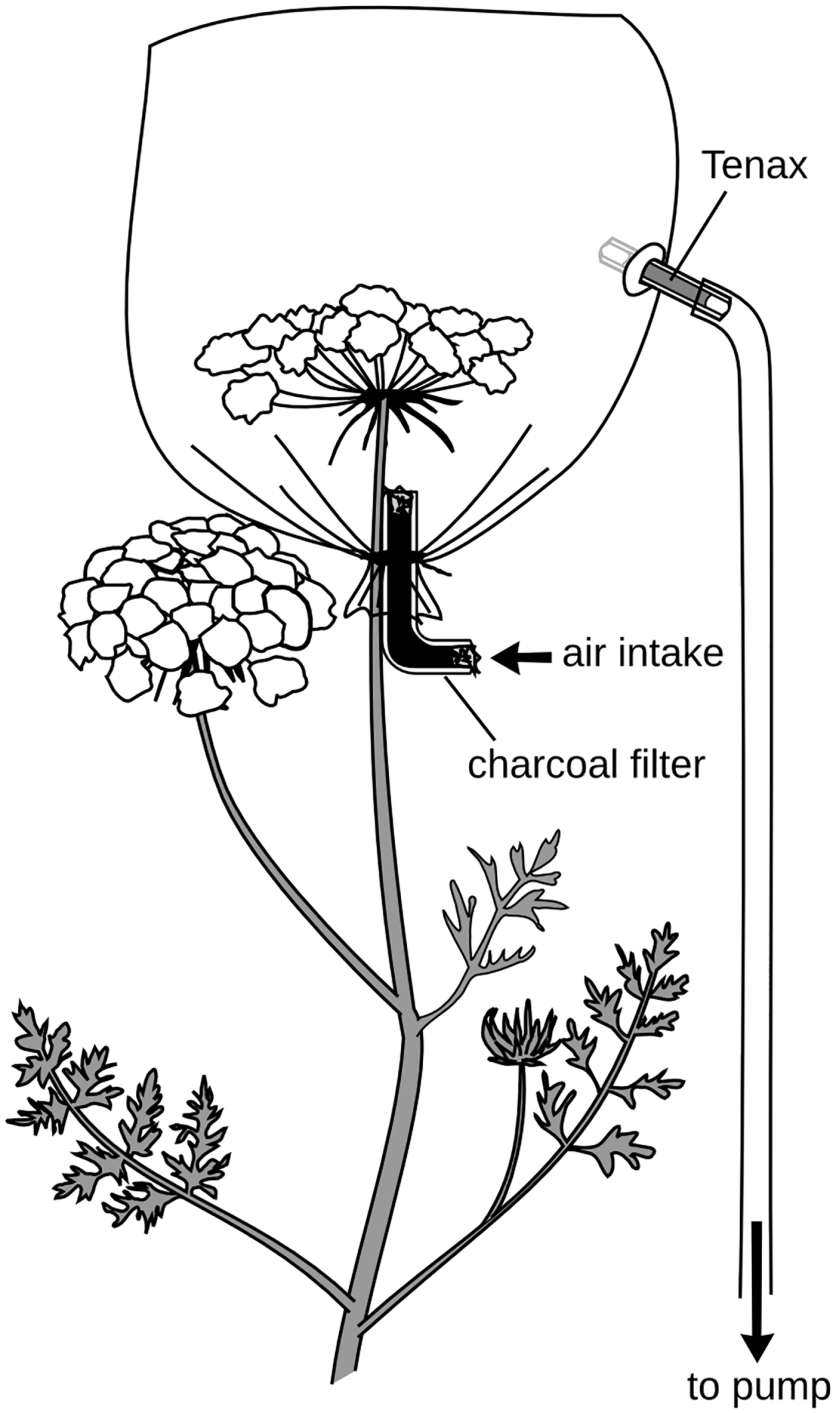
Apparatus for collecting carrot headspace volatiles.

Each headspace sample was collected *in situ* using the active sampling apparatus in Fig 1. The primary umbel of each flower was fitted with a nylon oven bag and, insofar as it was possible, leaf material was excluded from the bag. In order to ensure floral volatiles rather than green leaf or ambient compounds in the air were being collected, each set of collections included a control where the bag was secured around a leaf. Each bag was fitted with a charcoal filter at the base to remove ambient VOCs. We used a pump with an airflow rate of 500mL/min, split four ways so that 125mL/minof air was pulled through each headspace collection apparatus and into a Tenax^®^ filter, which adsorbed the floral VOCs. The Tenax^®^ filter was constructed from a 15mm long, 10mm diameter glass tube containing 60mg of Tenax^®^ 35/60 (Grace Davidson Discovery Sciences, VIC, Australia) held in place with silane-treated fiberglass (Grace Davidson Discovery Sciences, VIC, Australia). Tenax tubes were conditioned prior to use by heating for 3 hours at 250°C under a stream of nitrogen gas, and the charcoal filters were baked overnight at 150°C in a filtered-air oven. After VOC collection, each tenax was desorbed by solvent extraction with 1mL of n-hexane (Sigma-Aldrich, 99% purity).

To obtain quantitative values of each VOC identified in the headspace samples, we added an internal standard of nonadecane (10 μg) to the 1 mL of n-hexane (Sigma-Aldrich, 99% purity) used to elute the tenax tubes. A sample containing 10 μg/mL of each of the identified compounds was run as an external standard.

We used one microliter of each headspace extract for gas chromatography coupled with mass-spectrometry (GC/MS). The subsample was injected into a Varian 3800 gas chromatograph (Varian Walnut Creek, CA, USA) with the injector port set at 250°C, and then run through a DB5-MS non-polar column (J&W Scientific Folsom, CA, USA) with dimensions 30m x 0.25mm id x 0.25μm film thickness. The column was raised from 40°C up to 280°C at a rate of 4°C/min, and then held at 280°C for 5 minutes. We used a constant flow of helium as a carrier gas (1 mL/min). Injections were splitless for 36 seconds. A Saturn 2200 mass spectrometer (MS, Varian Walnut Creek, CA, USA) ionized the molecules from a mass range of 29 m/z to 399 m/z, after the GC separated each compound present in the extract according to their volatility. The abundance of each compound was then recorded as the area under each peak. After each sample was run, we identified compounds by comparing MS results to a database (NIST MS Search 2.2). Synthetic compounds were injected with the same GC-MS protocol to confirm identification.

To compare the compounds’ abundance in each extract, we used the internal standard method to calculate quantities, multiplying the known amount of the internal standard nonadecane (10 ng) by the area of the compound of interest and the response factor (measured from an external standard run containing a known quantity, 10 ng, of both nonadecane and the compound of interest), then dividing by the area of the internal standard. Volatile collections were done over a different time period for each treatment (variety: 24 hours, time: 3-5h), so for comparison of each compound across all the experiments, we calculated the emission rate by dividing the quantity of each peak by the amount of time of collection in order to obtain value in μg/h.

### Statistical analysis

Phenology data were recorded as a count variable—the number of days between seed sowing and flowering. Because all plants were kept under the same conditions prior to blooming, we could compare them in a generalized linear model (GLM). We used a gamma error distribution to account for the observed variance-mean relationship. The predictor variables were plant variety and plant line.

Nectar, pollen viability, and seed set data were collected over multiple days, creating the risk of temporal autocorrelation. As the temperature treatments were conducted in two locations each, there was also the potential for spatial autocorrelation within each glasshouse. In order to account for this variance and non-independence, we used generalized linear mixed-effect models (GLMMs) with date sampled and location as crossed random effects. We used the *lme4* library [48] in the R statistical programming language [49] to perform most of our analyses. We used the following model selection process to determine our best-fitting model. All permutations of the predictor variables (plant variety, temperature, and time-of-day, and their interactions) were used to create candidate models, and final models were selected if their AIC scores were within two points of the best-fit model; if multiple models met this criteria, we took a model average [50] using the *MuMIn* package 1.15–6 [51]. In order to obtain p-values for the final models, we used the Satterthwaite method of denominator synthesis, implemented within the *lmerTest* package [52]. It should be noted that this method calculates non-integer degrees of freedom. Where relevant, models were checked for over-dispersion (where error distributions were not Gaussian) or normality of residuals and homoscedasticity (for Gaussian models). In addition to differences in the mean response across treatments, initial examination of the data suggested that there may be differences in the variance of the response variables. To test for these differences, we used Levene’s test.

Seed set data were recorded as a count variable, as it was not possible to count the number of initial florets once the seed heads dried to establish a proportion. Plants that failed to set seeds introduced numerous zeros to the data set. While not over-dispersed, the dataset did not conform well to the Poisson distribution and so was analyzed with a zero-inflated negative binomial GLMM in R, with the *glmmADMB* package [53]. Predictor variables in the initial model were the pollen viability (as a covariate to control for the quality of pollen used in each hand-pollination), temperature at the time of pollination, plant variety, and time of day.

Pollen viability data were recorded as a binomial variable, where individual pollen grains were either viable or not. The proportion of viable and inviable pollen grains were tested in a GLMM with binomial errors and a logit link function. Because pollen stored at -80°C slowly loses viability over time [54; 55], we included the number of days between collection and processing as a fixed covariate in final model to account for the between-sample variation in storage time. Other predictor variables in the initial model were the plant variety and temperature and time of day at the time of pollen harvest. As this model was significantly over-dispersed, we also included individual sample as a random effect [56].

Nectar sugars were measured as the concentration of fructose and glucose; no sucrose was found. As the concentrations of the two sugars were tightly correlated (R^2^ = 0.983), only glucose was examined in a GLMM, with a gamma error distribution to account for the observed variance-mean relationship. Flowers varied in umbel diameter, which could influence the amount of nectar collected with the dipping methodology. Therefore, we controlled for flower size by adding the diameter of the umbel as a fixed covariate. Other predictor variables in the initial model were temperature at the time of nectar collection, plant variety, and time of day nectar samples were taken.

Nectar phenolics were expressed as concentrations. Three phenolic compounds were found in carrot nectar: caffeic acid, coumaric acid, and ferulic acid. As initial plots revealed that each compound reacted differently to different conditions, it was necessary to examine all three. To avoid multiple single-response models and to capture shifts in the combined phenolic bouquet, we conducted a nonmetric multidimensional scaling (NMDS) ordination within the R package *vegan* [57] with Bray-Curtis dissimilarity to account for the large differences in mean concentration. To test whether the three compounds varied across variety, temperature, and time of day, we used a permutation multivariate ANOVA (PERMANOVA) procedure (function '*adonis*') from the same package.

Floral volatiles were expressed as the concentrations of methyl salicylate, nonanal, and phenylacetaldehyde—the three compounds found in carrot floral volatiles which bees are able to sense (measured in previous work as a consistent electroantennograph response; S1 Fig). To account for the three compounds simultaneously, we ran a NMDS ordination for each of the volatile datasets with variety, temperature, and time of day as predictor variables. As with the phenolic data, we used a PERMANOVA procedure to test for significance. To aid with interpretation of these multivariate results, we then conducted univariate analyses on each volatile. Data exploration revealed that there was a non-linear relationship between time-of-day and volatile concentration, so we conducted a generalized additive mixed-effects model (GAMM), which allows for non-linear relationships between predictor and response variables [58]. In the GAMM, time sampled was a smooth term, while plant ID was a random effect. The amount of smoothing was determined in the model using maximum-likelihood within the *gamm4* package [59].

## Results

**Fig 2:**
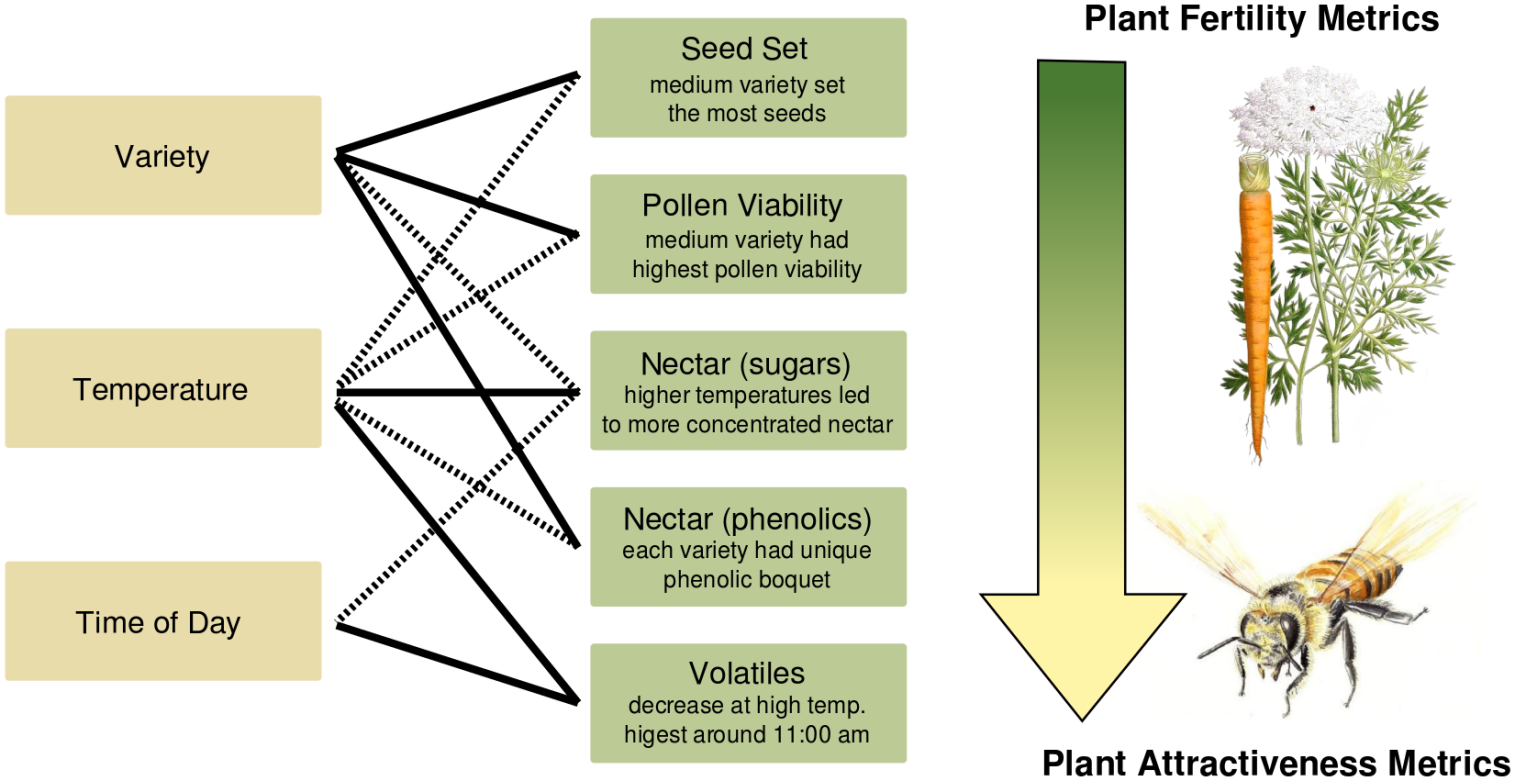
Relationships between different factors examined in this study. Solid lines indicate a statistically significant relationship. Dotted lines indicate factors that were conserved in the final selected models, but were not statistically significant. We did not find any significant interaction effects.

We found numerous effects of plant variety, temperature, and time-of-day on measures of both plant fertility and attractiveness to pollinators (see Fig 2 for a summary).

### Plant Fertility Metrics

#### Phenology

**Fig 3:**
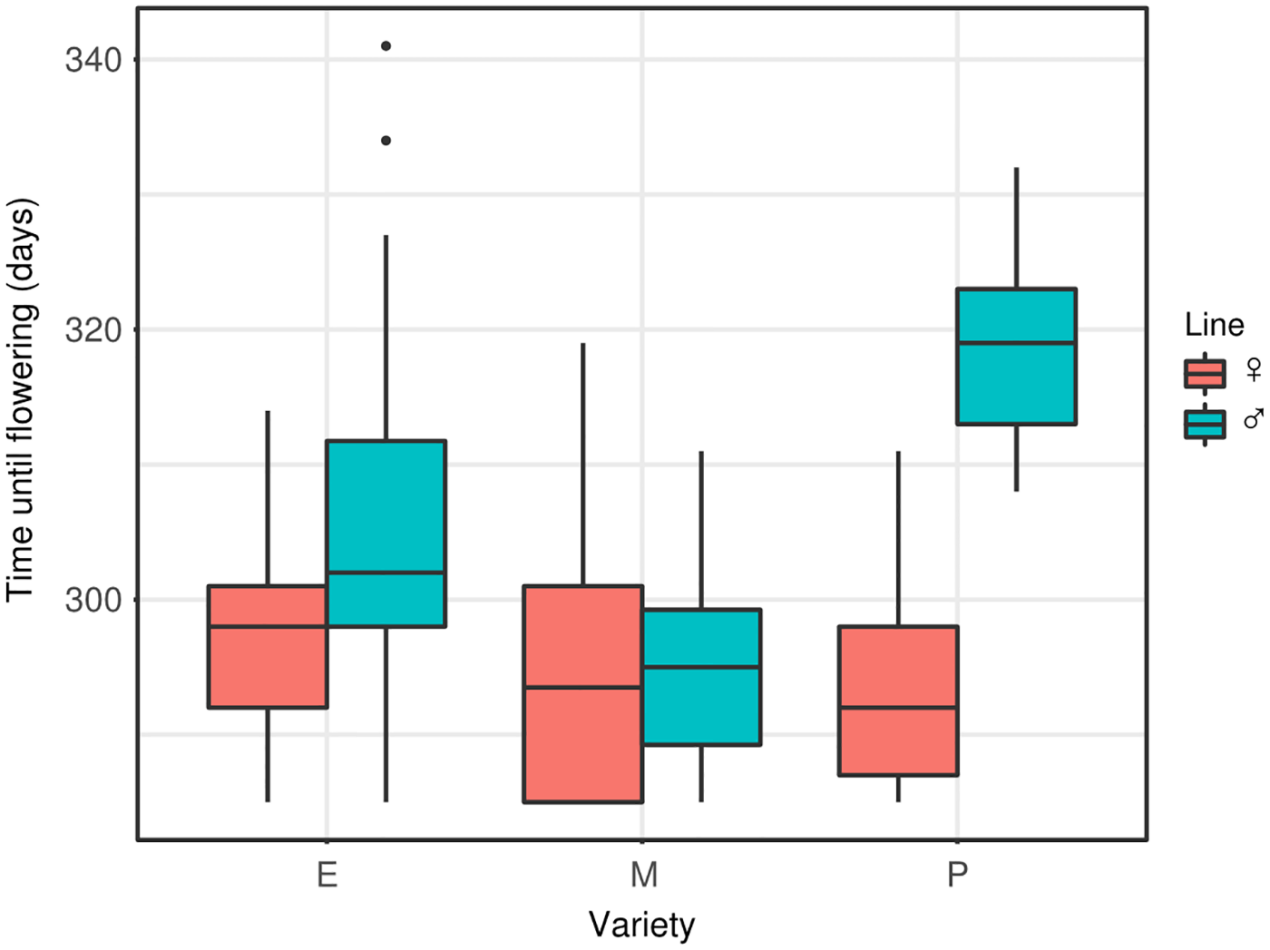
Number of days between seed sowing and flowering between the three carrot varieties. Variety: E = excellent, M = medium, P = poor. Line: male sterile (♀) and male fertile (♂) lines broken out for each variety. Data ceased being collected at 365 days. Boxes represent the middle 50% of the data, lines within boxes are the median.

A number of plants failed to flower at all: 5% of male-sterile and 22% of male-fertile carrots did not send up a flowering stalk after one calendar year. The rate for male-fertile plants was heavily influenced by the poor line, which accounted for 76% of failures (51% of these plants did not flower). Of the plants that did bloom, there was considerable spread in flowering time, with the first plant blooming on day 285 and the last on day 341. Male-fertile lines bloomed significantly later than male-sterile lines, and there was an interactive effect between variety and line, with the poor variety having the widest gap between the bloom time of the male fertile and male-sterile lines (Fig 3, Table 1). The male-sterile excellent variety had the most tightly grouped flowering time (P < 0.001; F = 8.560; Levene's test for differences in variance)

**Table 1:** Coefficients table of GLM for carrot phenology. Variety: E = excellent, M = medium, P = poor. Line: male sterile (♀) and male fertile (♂). The intercept condition is the male-sterile, excellent line.

	Estimate	SE	t statistic	P value
intercept	3.373 x 10^−3^	8.855 x 10^−5^	380.908	< 0.001 ***
Variety (M)	2.675 x 10^−5^	1.274 x 10^−5^	2.099	0.036 *
Variety (P)	4.577 x 10^−5^	1.267 x 10^−5^	3.611	< 0.001 ***
Line (♂)	-9.521 x 10^−5^	1.345 x 10^−5^	-7.076	< 0.001 ***
Variety (M): Line (♂)	8.971 x 10^−5^	1.945 x 10^−5^	4.612	< 0.001 ***
Variety (P): Line (♂)	-1.783 x 10^−4^	2.102 x 10^−5^	-8.483	< 0.001 ***

Significance codes:

* < 0.05,

** <0.01

*** <0.001

#### Seed set

**Fig 4:**
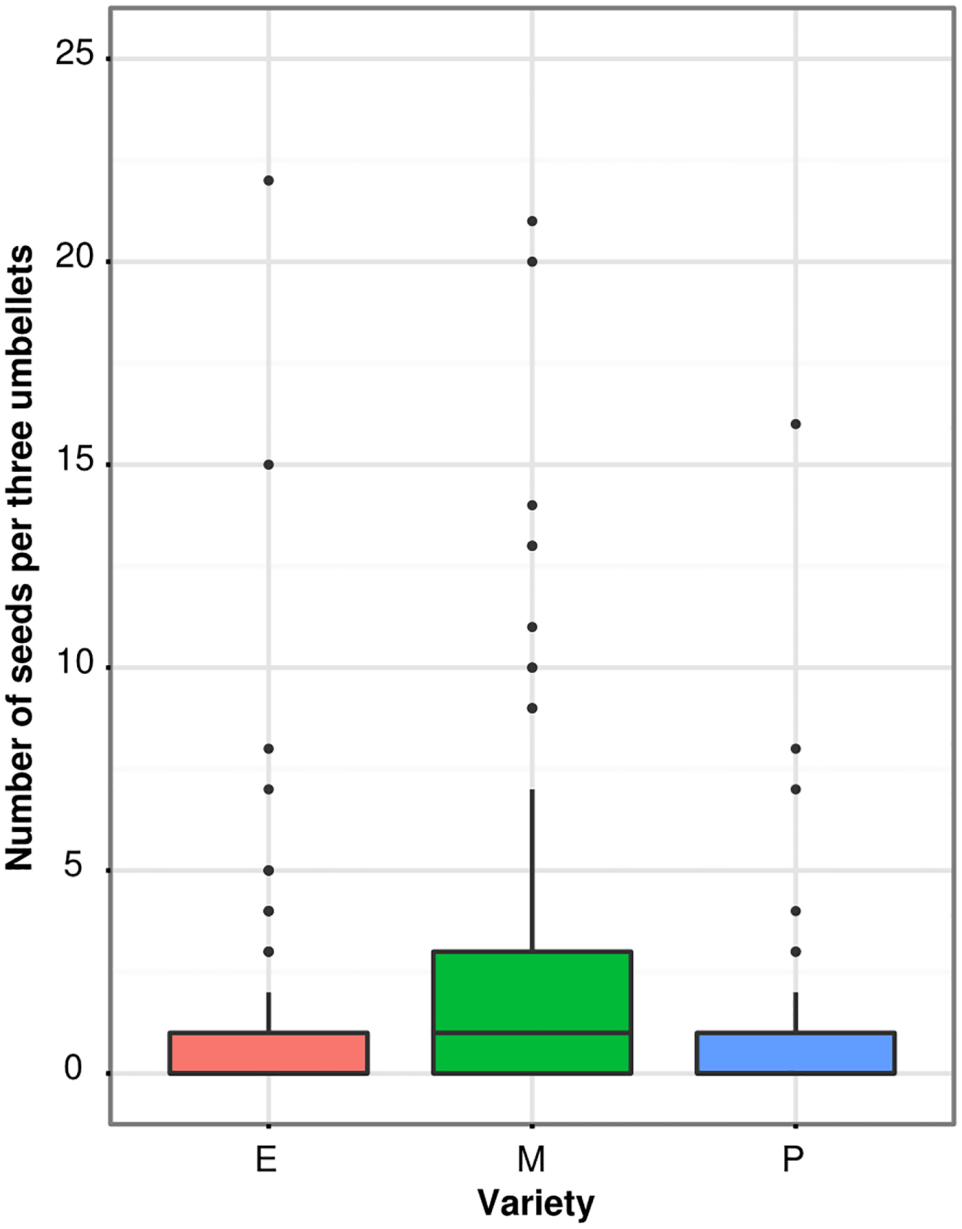
Seed set amongst the three carrot varieties. E = excellent, M = medium, P = poor. Boxes represent middle 50% of data, lines within boxes are the median.

The rate of seed set was low, with fewer than half of the hand-pollinated umbels setting seed. This may have been due to poor weather, poor pollen viability, and, potentially, the presence of the brown shield bug (*Dictyotus caenosus* (Westwood), Hemiptera:Heteroptera), which was able to enter the seed heads through the exclusion mesh. Nearly every mesh pollinator exclusion bag contained at least one *D*. *caenosus*, and though, to our knowledge, there is no record of *D*. *caenosus* feeding on carrot seed, we cannot exclude the possibility that this generalist plant-feeder used the seed heads as a food source. In total, less than half of the hand-pollinated umbels set seed. Despite the overall low seed set, we still found differences between the carrot varieties, such that the medium-performing variety set significantly more seed than the other two varieties (Fig 4), with an average of 1 additional seed per three umbellets (P = 0.005; z = 2.788; GLMM). Temperature (P = 0.995; z = 0.010; GLMM) and proportion of viable pollen (P = 0.383; z = 0.872; GLMM) were both retained in the best-fitting model, though neither was a significant predictor of seed set. The medium variety was also more variable in seed set than the other two varieties (P = 0.001; Levene's test), as would be expected for count data, where the variance often increases with increasing means.

#### Pollen viability

**Fig 5:**
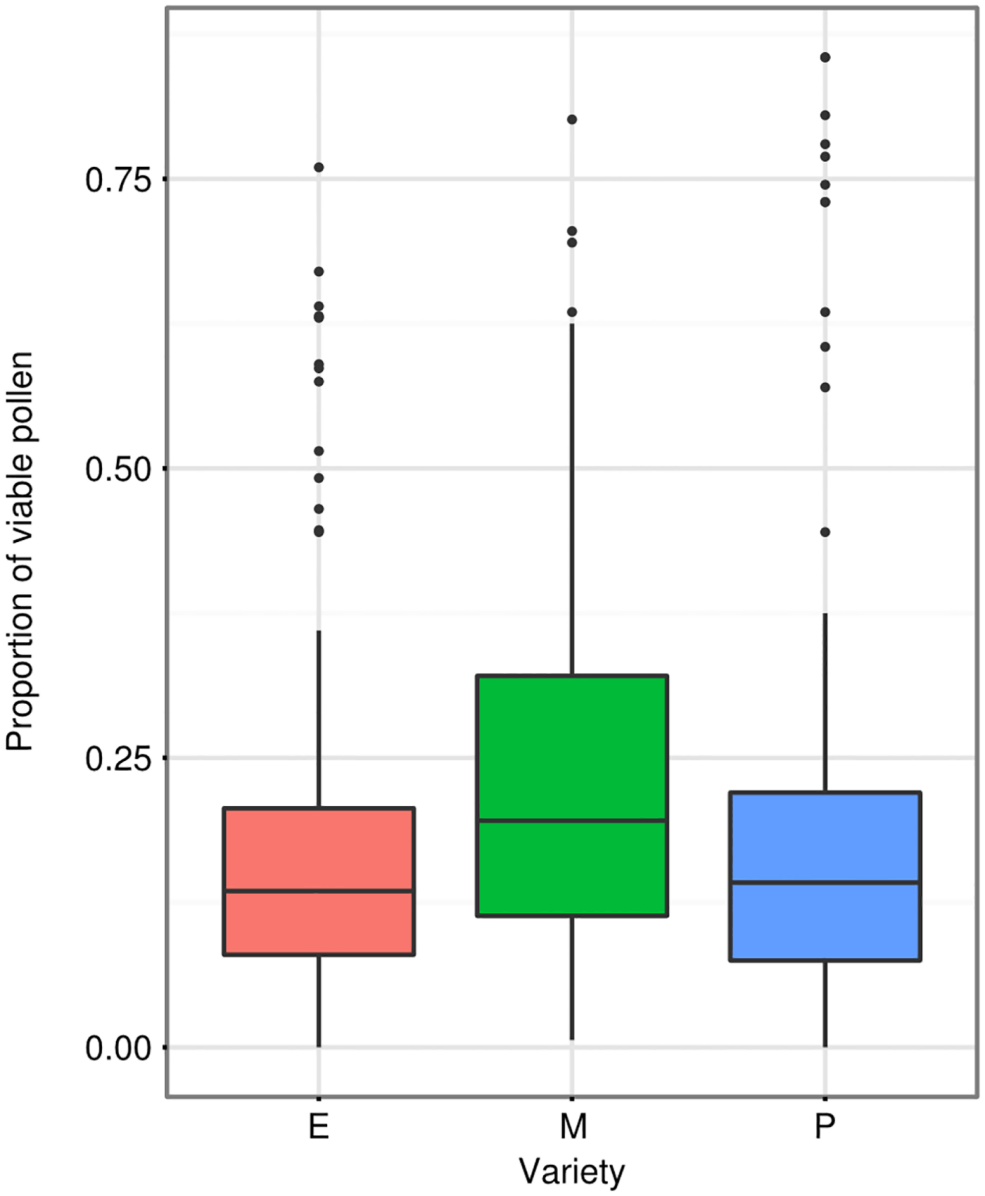
Pollen viability amongst the different varieties. Each sample point represents all male-fertile flowers with pollen at the time of sampling (evenly spread throughout the 7 time and 3 temperature treatment combinations). Viability was calculated as a proportion viable out of 200 grains. E = excellent, M = medium, P = poor. Boxes represent middle 50% of data, lines within boxes are the median.

Overall pollen viability was low, with a median of 14.5%. This is comparable to previous estimations of carrot pollen viability in New Zealand (S1 Text), but is low compared to the viability of cultivated carrot pollen elsewhere in the world [21; 43; 60], and much lower than wild carrot pollen (~80%) [35]. Temperature was retained in the final model, but was not a significant predictor of viability (P = 0.825; z = 0.22; GLMM). There was a significant difference in pollen viability between varieties (Fig 5; P = 0.004; z = 2.866; GLMM), with the medium variety being the highest, about 45% higher than either of the other two (observed mean of 21.2% versus 14.3% for excellent and 15.9% for poor). The medium variety was also more variable than either the poor- or excellent-performing varieties (P = 0.023; Levene's test).

### Plant Attractiveness Metrics

#### Nectar quality

**Fig 6:**
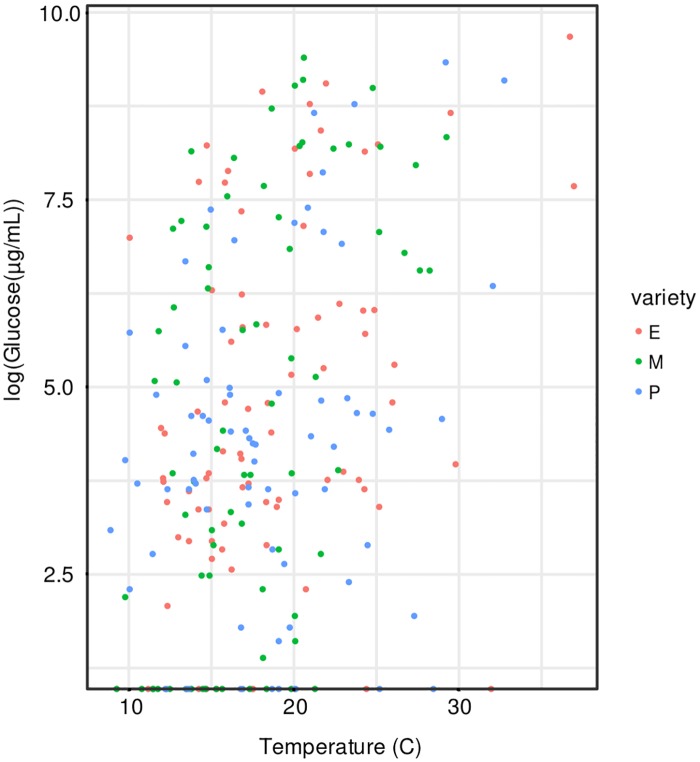
Log glucose concentration in nectar at different temperatures for the female lines of all three carrot varieties. E = excellent, M = medium, P = poor.

**Fig 7:**
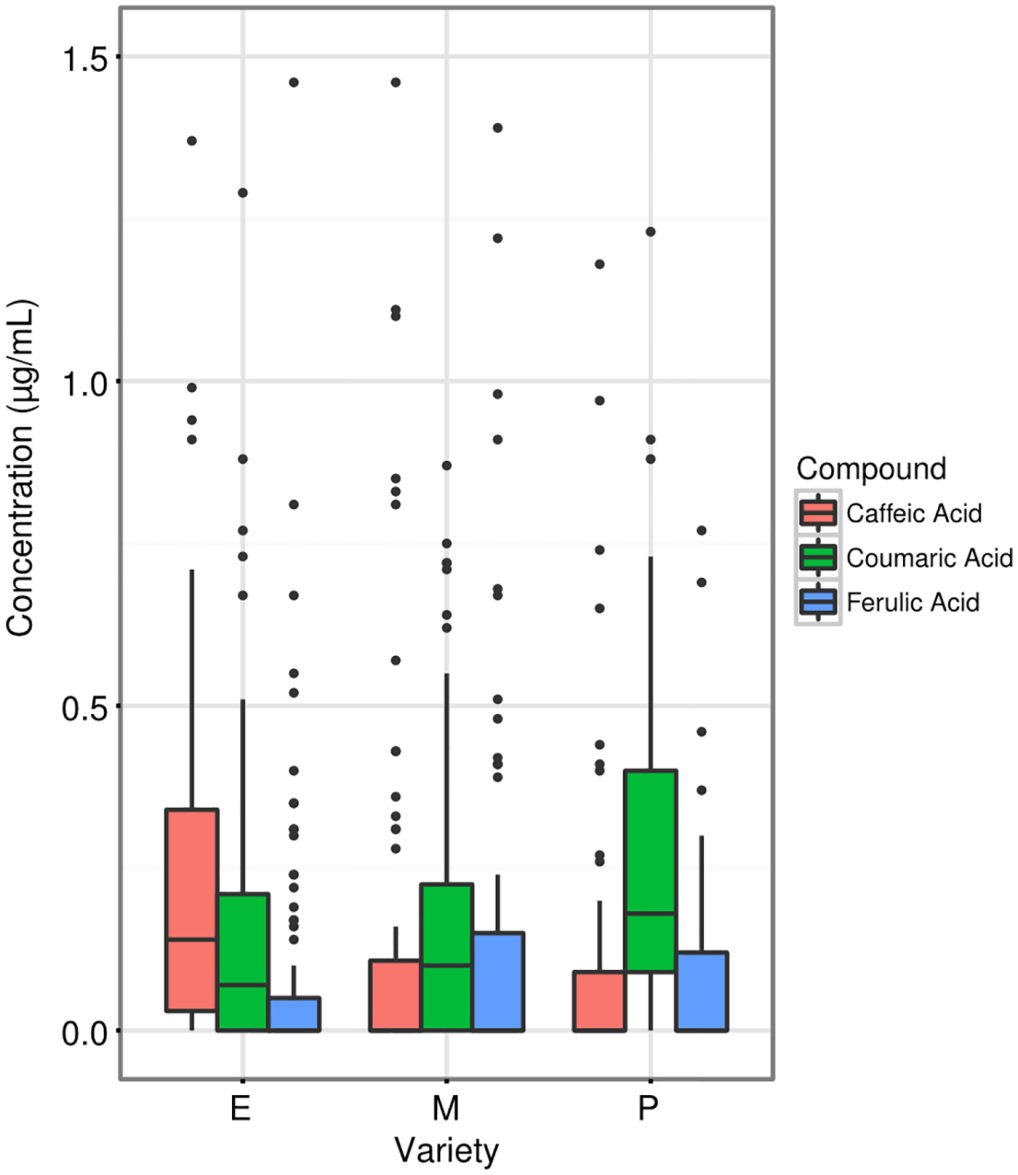
Concentrations of nectar phenolic compounds by carrot variety. E = excellent, M = medium, P = poor. Boxes represent middle 50% of data, lines within boxes are the median.

Glucose and fructose were found in a close to 1:1 ratio (1.019:1; R^2^ = 0.983; LM). No sucrose was detected. There was a significant effect of temperature (P = 0.002; t = 3.091; GLMM) on glucose concentrations, with increasing temperature being correlated with higher concentrations of sugars (Fig 6). Time-of-day, variety, and an interactive effect between time-of-day and variety were all retained in the final model, though none of them were significant predictors of sugar concentration. For phenolic compounds in the nectar, each of the three varieties had a different composition (P = 0.012; F = 3.393; PERMANOVA), with the excellent variety having high concentrations of caffeic acid, moderate concentrations of coumaric acid, and low concentrations of ferulic acid; the medium variety had low concentrations of caffeic acid, moderate concentrations of coumaric acid and moderate concentrations of ferulic acid; the poor variety had low concentrations of caffeic acid, high concentrations of coumaric acid and moderate concentrations of ferulic acid (Fig 7). The excellent variety was more variable in its concentration of caffeic acid than the other two varieties (P = 0.017; Levene's test). Temperature was not a significant predictor of nectar phenolic composition (P = 0.080; F = 2.566), but was retained in the final model.

#### Floral volatiles

**Fig 8:**
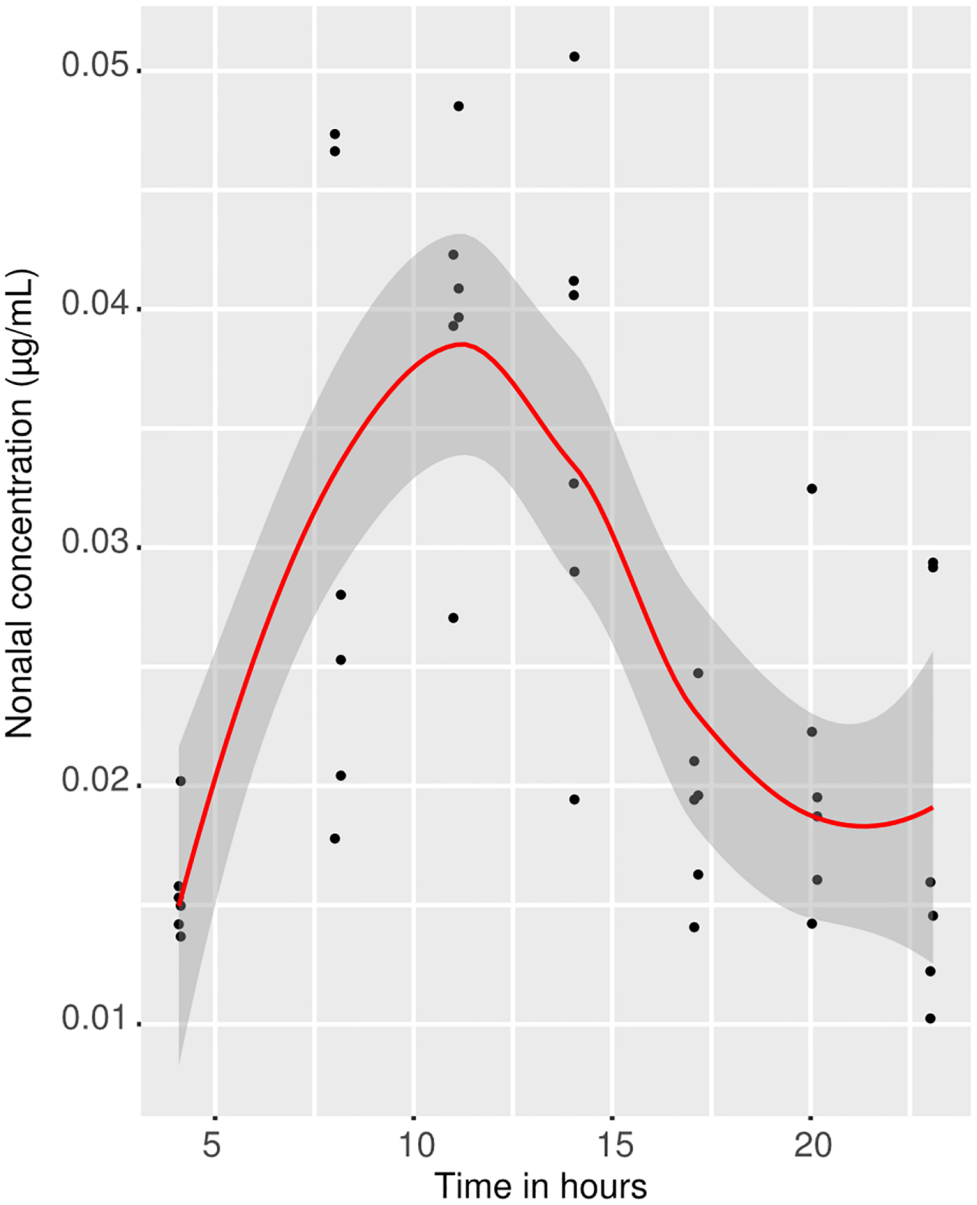
Concentration of nonanal versus time-of-day. Values are from six 'medium' plants sampled repeatedly over the course of 24 hours. The red line is the trend line created by the GAMM, the dark grey area is the 95% confidence interval.

Of the three compounds contained in the male-sterile carrot flowers' floral bouquets that honey bees are capable of sensing, only nonanal was present in all samples. There was no effect of variety on floral bouquet (P = 0.671; F = 0.467; PERMANOVA), but there was a significant effect of temperature (P = 0.028; F = 4.743; PERMANOVA), with higher temperatures corresponding to lower volatile emissions. As plants were tracked through the course of a 24-hour day, there was a significant spike in nonanal concentration at around 11:00am (Fig 8; P < 0.001; t = 17.460; GAMM), just prior to the afternoon heat. Although there was no significant effect of variety, the medium variety was more variable than the other two (P = 0.009; Levene's test), meaning that the significant time-of-day result despite this background variability, which was tested using the medium variety, is likely robust.

## Discussion

While environmental conditions at flowering time affected some plant fertility and attractiveness measures, we did not find any interaction effects between plant variety and temperature. This implies that the differences observed between the poor-, medium-, and excellent-performing carrots were due largely to innate plant characteristics (Fig 2), which did not respond to temperature. Our data suggest numerous mechanisms of poor performance in the field, as the 'poor' variety consistently underperformed the other two: it bloomed late, 50% of the male-fertile plants failed to even initiate flowering, had the worst synchronization between male-sterile and male-fertile lines (Fig 3), relatively low seed set (Fig 4), and pollen viability typically below 20% (Fig 5). Additionally, the nectar phenolic profiles showed a wide gap between the varieties. The poor line's nectar was high in coumaric acid, which has been found to upregulate bee detoxification pathways [61], and ferulic acid (Fig 7), which, while commonly found in honey bee propolis [62], is thought to be an insect feeding deterrent [63]. Similarly, the poor line is low in caffeic acid, which is highly attractive to bees at modest concentrations [64].

Underperformance in multiple categories, from purely physiological characters to factors which influence plant attractiveness to pollinators, is a satisfying explanation for why a particular variety is observed to have low yield, but we have found that there is not a similarly easy explanation for the high yield of the best-performing variety. In fact, the excellent variety was observed to perform worse than the medium variety under controlled conditions: the excellent variety had somewhat worse synchronization between the male-sterile and male-fertile lines than the medium variety, and seed set and pollen viability on par with the poor variety. The excellent variety, however, distinguishes itself in its nectar phenolics, where it has high concentrations of the attractive caffeic acid, and is more consistent overall, with lower variances than the medium line in nearly every aspect, including bloom duration of the male-sterile line. Although there may be other differences that we did not capture, these two may result in increased pollinator attractiveness and better conditions for those pollinators to cross-pollinate the hybrid lines under field conditions.

### Implications for industry

Our results are particularly important as previous studies have identified poor pollination as a major cause of low seed yields in hybrid carrot [18; 43; 65]. Varieties that are better able to attract pollinators may be better able to fare annual fluctuations in pollinator populations as they could draw in pollinators from the surrounding environment, a trend already observed in mass-flowering crops [66]. The importance of pollinator attractiveness for determining which varieties succeed or fail is magnified here by the very low pollen viability of these commercial hybrid carrot lines—typically less than 30%. If viability rates were closer to wild carrot (~80% [35]), the required pollen deposition would be reduced by ½ to ¾, and thus pollination could be achieved with fewer insect visits and this would reduce the effect of differential attractiveness between the varieties.

We might join the numerous other authors that encourage the breeding of crops for increased insect attractiveness [10; 18], or higher capacity for seed production [67], or suggest that pollen viability be selected for [43]. However, it is important to keep in mind that vegetable seed crops are not bred for seed production, they are bred for the desirable characteristics of the plants raised from that seed. Indeed, these two goals are often at odds with each other. For example: an onion that produces two flower spikes will produce much more seed, but it will also produce low-grade onions with doubled hearts [68]. The result of this compromise between seed set and plant characters has been hybrid production systems, which produce vigorous, uniform progeny, but may set less seed than open pollinated systems, if for no other reason than some portion of the field must be occupied by the pollinizer line, from which seed is not collected. As a result, hybrid carrots often yield less than 50% of the seed produced by open-pollinated carrots [10; 32].

Although the yield of hybrid carrot seed is likely to remain lower than open pollinated seed, there is obviously latitude for higher yield. In our experiment, synchrony of bloom was poor, as is common with hybrid crops [43; 69; 70]. Male-sterile plants of all three varieties began blooming before the male-fertile line, meaning that some primary umbels, which typically have high seed yield [42], would have failed to set seed due to absence of available pollen. One cultural solution already underway in industry is to cut carrot plants early in the season to delay the flowering of one of the lines in order to synchronize bloom, which then increases yield [18; 43]. Cutting the plants at an early stage of flowering has been shown to delay bloom by 10–14 days [18]—however, this means that growers would need multiple cuttings to successfully synchronize poor male-fertile and male-sterile lines. Planting the male-fertile line even one month earlier than the male-sterile line isn't enough to fully align the two (South Pacific Seeds, Methven, New Zeland, pers. comm).

Another cultural solution is to increase the number of pollinators in the field. Carrot has a generalist flower type [10; 30] and is visited by hundreds of insect species [28–31; 71; 72], many of which can contribute significantly to successful pollination [31; 43; 71; 73]. Honey bees have traditionally been used to pollinate the crop with a stocking density of 5–8 hives/ha in New Zealand [37; 74] and Australia [43]. This is considerably lower than the hive density used in the United States, which has stocking densities 2–4 times that (15–20 hives/ha [75]). Increasing the stocking density of honey bees may improve yield, but the discrepancy may represent the different pollinator communities in the two localities as honey bees do not appear to favor carrot as a forage source [10] and preferentially visit other attractive floral resources where possible [33]. Developing practices in which increase the numbers of other species shown to efficiently pollinate carrot, such as *Megachile rotundata* [76] (Hymenoptera), *Calliphora vicina* [77], and *Eristalis tenax* [78] (Diptera) may prove better options for New Zealand, though additional field trials would be necessary to verify a benefit.

In addition, there may be some room for improvement of the carrot varieties. Surprisingly, none of the cultivars we examined contained any detectable quantities of sucrose, which is much more attractive to bees than glucose or fructose alone [79; 80], and has been detected in hybrid carrot varieties in other parts of the world [17; 31]. Additionally, all three cultivars examined in this study have very low pollen viability (<30%) compared to elsewhere in the world (~50% [21; 43; 60]). Inbreeding depression has been observed for numerous other agronomically important traits in carrot [67], and it may be the case that New Zealand hybrid carrots have poor pollen viability because of this. However, there is considerable genetic diversity within cultivated carrot globally [81]—including within groups sharing the same agronomic characters [75]. This being the case, introducing breeding stock from elsewhere in the world may alleviate some of the stress in the New Zealand hybrid carrot production system while still selecting for marketable qualities, and the added genetic variability could result in varieties more robust to changes in climate and weather patterns. Although hybrid seed crops are not typically bred for the fitness of the parent lines, it may become necessary to do so if poor plant vigor, poor attractiveness, lower pollinator populations, and increased stress from a warming climate lead to seed sets much lower than they are today.

### Implications for pollination under climate change

In the carrot seed producing region of New Zealand, climate change is forecast to decrease rainfall and increase surface temperature, leading to seasonal shifts and an increase in droughty conditions [38]. Climate change has been linked to negative crop plant outcomes, including: increased susceptibility to insects and disease [82; 83], decreased competitiveness versus weeds [82; 84], decreased effectiveness of herbicides on weed control [84; 85], reduced overlap between bloom and pollinators [85], reduced pollen viability [22; 23], changes in volatile emissions [86], and quantity and quality of nectar which may affect plant attractiveness [27; 87]. Previous work in New Zealand has identified that higher temperatures may result in increased foraging by honey bees, while reducing species richness [88]—primarily native and introduced flies. As a recent meta-analysis has found that crop yields tend to increase with pollinator richness, independent of honey bee abundance [89], this may result in an increased number of visits, but a decrease in average visit quality.

We found that there was an effect of temperature on nectar concentration and volatile emission, but no other plant characteristics in our study, although temperature was retained in the models for seed set and pollen viability (Fig 2), meaning it added explanatory power. It is important to remember that we attempted to expose plants only at the pollen formation stage during flowering, rather than throughout development. Temperature has been shown to affect plant development at numerous critical periods [22], and exposure to high temperatures at an earlier point may have reduced plant vigor beyond the effects we observed here. However, the temperatures we exposed plants to in the hot treatment are slightly higher than the future temperature projections for the region [38], so there are unlikely to be further effects on pollen viability in a warmer climate, which is fortunate given the already low viability of the hybrid varieties. Hybrid crops such as carrot are highly susceptible to pollination disruption, due to their requirement for pollen transmission across pollinizer lines. Therefore, if a warming climate leads to fewer non-managed pollinators, this could potentially reduce yield. Given carrot's already modest attractiveness to pollinators compared with weedy species [10; 33], the potential increase in nectar concentration to above the attractive range of 30–50% [25] and change floral scent could further limit its competitiveness. The situation may be exacerbated by the warming-induced increase in weed vigor predicted by other studies [82–84], as it would increase competition for a more limited pool of pollinators, with a net negative effect on seed set.

### Conclusions

The combination of lowered attractiveness with higher competition for pollinators and higher losses to weeds could prove to be a difficulty for future hybrid carrot seed production. If other carrot seed growing regions of the world experience a decline in unmanaged pollinators, as New Zealand is expected to, it could lead to a fragile production system through over-reliance on honey bees [89; 90]. As insurance against adverse pollination conditions, future hybrid production systems may have to balance agronomic traits with the plant's ability attract to pollinators and set seed or, potentially, domesticate currently unmanaged pollinator species.

## Supporting information

S1 Fig Traces from the flame ionization detector (FID) of the gas chromatograph (top line) coupled with the electro-antennogram detector (EAD) responses (bottom line) from a honey bee antenna. Three electrophysiological responses were detectected from the EAD trace, circled in red: phenylacetaldehyde, nonanal, methyl salicylate, from left to right. The antenna was exposed to a carrot flower headspace sample that had been collected over a period of 24 hours.
(TIF)

S2 Fig Temperatures experienced by each shadehouse and glasshouse treatment. Histogram of temperatures collected at chest height from data loggers (onset HOBO Prov2 temp/RH meters) from the two enclosures for each of the three temperature treatments during the course of the experiment.
(TIF)

S3 Fig Stages of development in carrot florets. Petals and anthers were removed from florets for photography.
(TIF)

S1 Table Coefficients table of GLM for flower phenology. Relationship between time between sowing and blooming and the plant variety, and the plant line (male sterile vs. male fertile), with interactions and a gamma distribution. The intercept condition is the excellent variety, male sterile.
(DOCX)

S2 Table Coefficients table of zero-inflated binomial GLMM for seed set. The final model retained temperature at the time of pollination, plant variety, and pollen viability as predictors of observed seed set per three umbellets. The intercept condition is the excellent variety.
(DOCX)

S3 Table Coefficients table of binomial GLMM for pollen viability. The final model retained temperature at the time of pollination, plant variety, and the number of days pollen was stored prior to processing as predictors of observed pollen viability in male fertile lines. The intercept condition is the excellent variety.
(DOCX)

S4 Table Coefficients table of LM for nectar glucose:fructose ratio. The intercept condition is nectar glucose (μg) per ½ umbel.
(DOCX)

S5 Table Coefficients table of GLMM for nectar sugar composition. The final model retained time-of-day, temperature at the time of pollination, plant variety, and the interaction between time-of-day and variety. The intercept condition is the excellent variety at the peak nectar emission time of 11:00am.
(DOCX)

S6 Table Coefficients table of ADONIS for nectar phenolic bouquet. The final model retained plant variety, temperature at the time of pollination, and time-of-day.
(DOCX)

S7 Table Coefficients table of ADONIS for floral volatiles; variety trial. (DOCX)

S8 Table Coefficients table of ADONIS for floral volatiles; temperature trial. (DOCX)

S9 Table Coefficients table of ADONIS for floral volatiles; time-of-day trial. (DOCX)

S1 Text Preliminary carrot pollen viability sampling method and results. (DOCX)

S1 Data Raw data from which analyses were generated. (CSV)
