# Supplementary figures and images for: Possible mechanisms of pollination failure in hybrid carrot seed and implications for industry in a changing climate
Source: PLoS One. 2017 Jun 30;12(6):e0180215. doi: 10.1371/journal.pone.0180215 (PMC5493370; doi:10.1371/journal.pone.0180215)

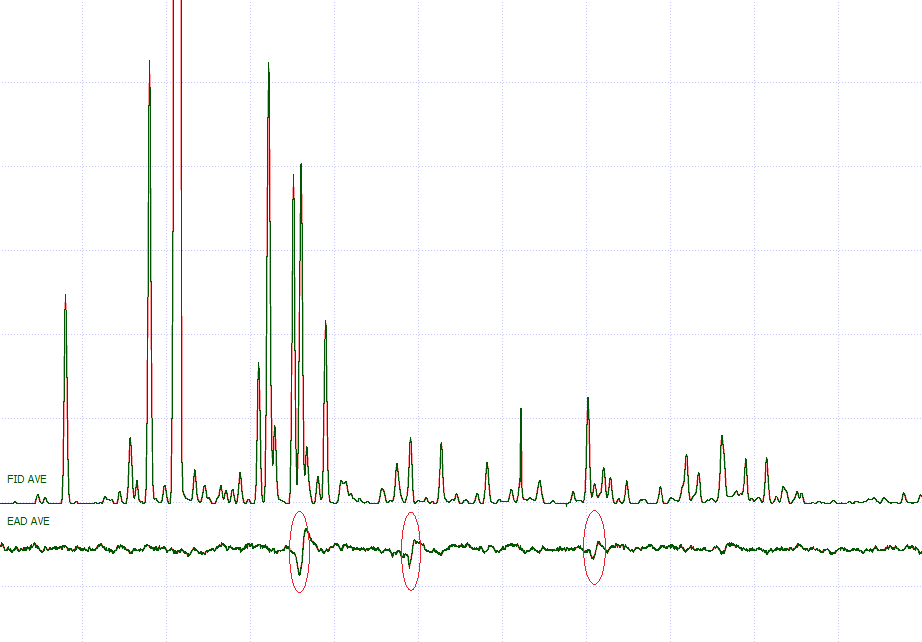

Supplement: S1 Fig — Three electrophysiological responses were detectected from the EAD trace, circled in red: phenylacetaldehyde, nonanal, methyl salicylate, from left to right. The antenna was exposed to a carrot flower headspace sample that had been collected over a period of 24 hours. (TIF) [file pone.0180215.s001.tif]

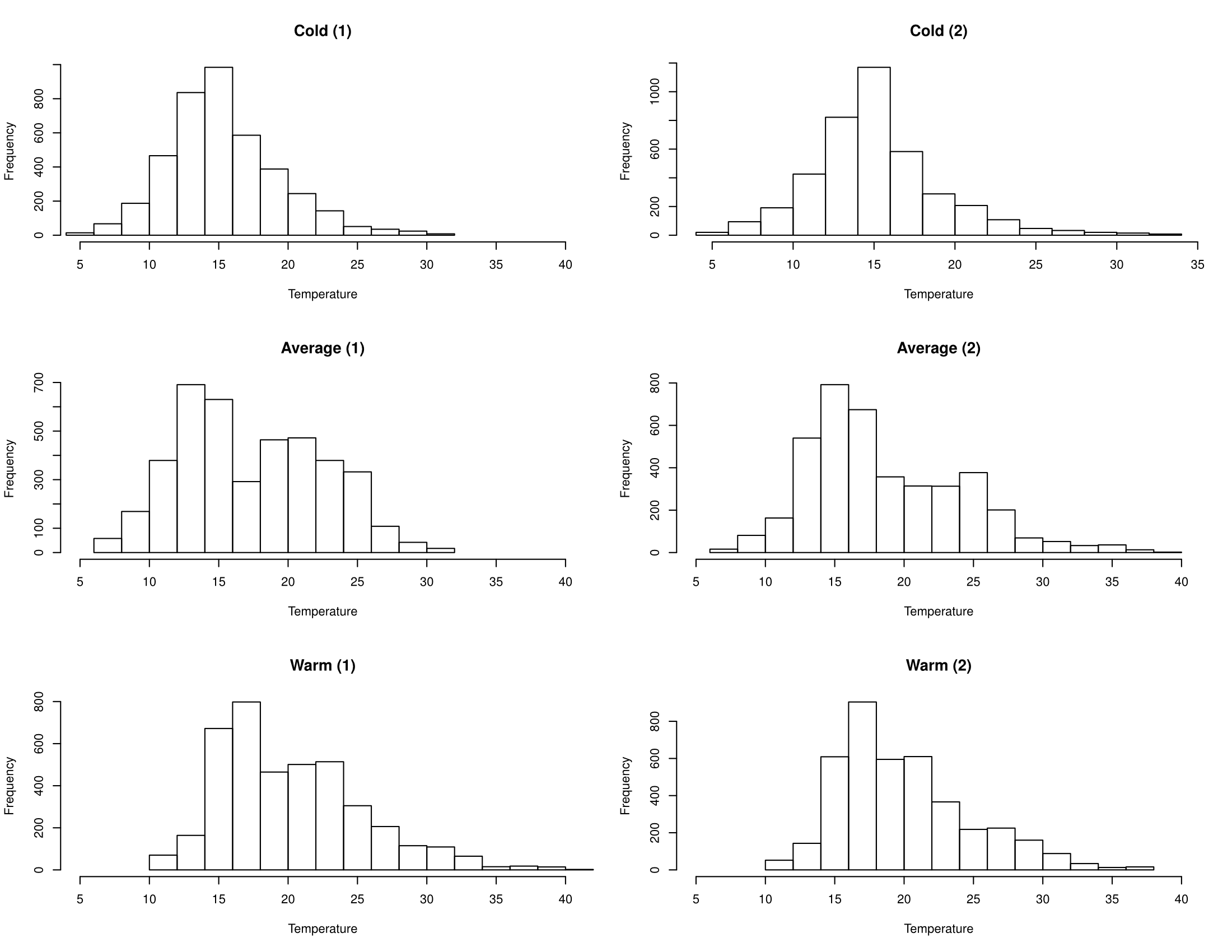

Supplement: S2 Fig — Histogram of temperatures collected at chest height from data loggers (onset HOBO Prov2 temp/RH meters) from the two enclosures for each of the three temperature treatments during the course of the experiment. (TIF) [file pone.0180215.s002.tif]

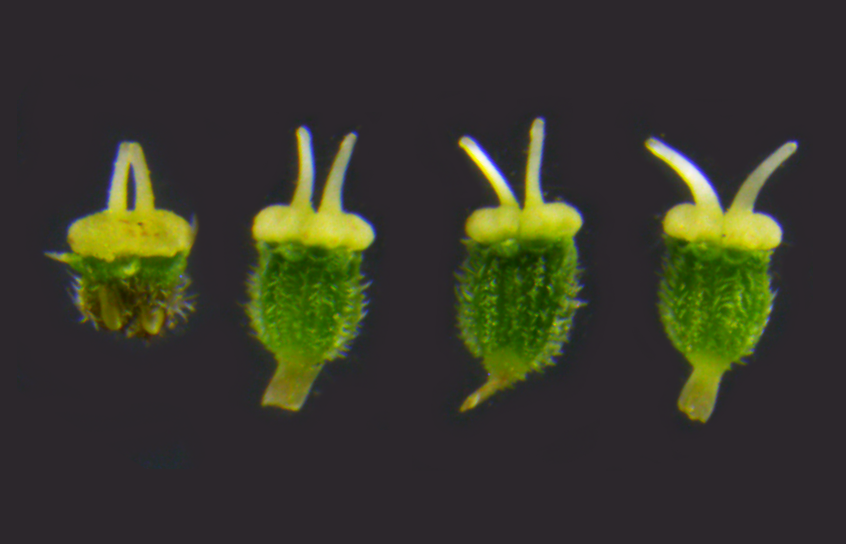

Supplement: S3 Fig — Petals and anthers were removed from florets for photography. (TIF) [file pone.0180215.s003.tif]
