# Supplementary material for: Possible mechanisms of pollination failure in hybrid carrot seed and implications for industry in a changing climate
Source: PLoS One. 2017 Jun 30;12(6):e0180215. doi: 10.1371/journal.pone.0180215 (PMC5493370; doi:10.1371/journal.pone.0180215)
Supplement: S1 Table — Relationship between time between sowing and blooming and the plant variety, and the plant line (male sterile vs. male fertile), with interactions and a gamma distribution. The intercept condition is the excellent variety, male sterile. (DOCX) [file pone.0180215.s004.docx]

**S1 Table. Coefficients table of GLM for flower phenology.** Relationship between time between sowing and blooming and the plant variety, and the plant line (male sterile vs. male fertile), with interactions and a gamma distribution. The intercept condition is the excellent variety, male sterile.

|  | Estimate | SE | t value | P value |
| --- | --- | --- | --- | --- |
| intercept | 3.373 x 10^-3^ | 8.855 x 10^-6^ | 380.908 | < 0.001 *** |
| Variety (medium) | 2.675 x 10^-5^ | 1.274 x 10^-5^ | 2.099 | < 0.001 *** |
| Variety (poor) | 4.577 x 10^-5^ | 1.267 x 10^-5^ | 3.611 | 0.036 * |
| Line (male fertile) | -9.521 x 10^-5^ | 1.345 x 10^-5^ | -7.076 | < 0.001 *** |
| Variety (medium) :  Line (male fertile) | 8.971 x 10^-5^ | 1.945 x 10^-5^ | 4.612 | < 0.001 *** |
| Variety (poor) :  Line (male fertile) | -1.783 x 10^-4^ | 2.102 x 10^-5^ | -8.483 | < 0.001 *** |

Significance codes: * < 0.05, ** <0.01 *** <0.001
