# Supplementary material for: Possible mechanisms of pollination failure in hybrid carrot seed and implications for industry in a changing climate
Source: PLoS One. 2017 Jun 30;12(6):e0180215. doi: 10.1371/journal.pone.0180215 (PMC5493370; doi:10.1371/journal.pone.0180215)
Supplement: S2 Table — The final model retained temperature at the time of pollination, plant variety, and pollen viability as predictors of observed seed set per three umbellets. The intercept condition is the excellent variety. (DOCX) [file pone.0180215.s005.docx]

**S2 Table. Coefficients table of zero-inflated binomial GLMM for seed set.** The final model retained temperature at the time of pollination, plant variety, and pollen viability as predictors of observed seed set per three umbellets. The intercept condition is the excellent variety.

|  | Estimate | SE | z value | P value |
| --- | --- | --- | --- | --- |
| intercept | 0.370 | 0.366 | 1.007 | 0.3138 |
| Pollen Viability | -0.852 | 0.974 | 0.872 | 0.3833 |
| Variety (medium) | 0.973 | 0.347 | 2.788 | 0.0053 ** |
| Variety (poor) | -0.319 | 0.362 | 0.877 | 0.3804 |
| Temperature | 1.118 x 10^-4^ | 0.011 | 0.010 | 0.9918 |

Significance codes: * < 0.05, ** <0.01 *** <0.001
