# Supplementary material for: Possible mechanisms of pollination failure in hybrid carrot seed and implications for industry in a changing climate
Source: PLoS One. 2017 Jun 30;12(6):e0180215. doi: 10.1371/journal.pone.0180215 (PMC5493370; doi:10.1371/journal.pone.0180215)
Supplement: S3 Table — The final model retained temperature at the time of pollination, plant variety, and the number of days pollen was stored prior to processing as predictors of observed pollen viability in male fertile lines. The intercept condition is the excellent variety. (DOCX) [file pone.0180215.s006.docx]

**S3 Table. Coefficients table of binomial GLMM for pollen viability.** The final model retained temperature at the time of pollination, plant variety, and the number of days pollen was stored prior to processing as predictors of observed pollen viability in male fertile lines. The intercept condition is the excellent variety.

|  | Estimate | SE | z value | P value |
| --- | --- | --- | --- | --- |
| intercept | -1.646 | 0.257 | 6.391 | < 0.001 *** |
| Days Stored | -0.006 | 0.002 | 2.506 | 0.012 * |
| Variety (medium) | 0.458 | 0.159 | 2.866 | 0.004 ** |
| Variety (poor) | -0.012 | 0.204 | 0.057 | 0.954 |
| Temperature | 0.001 | 0.006 | 0.220 | 0.825 |

Significance codes: * < 0.05, ** <0.01 *** <0.001
