# Supplementary material for: Possible mechanisms of pollination failure in hybrid carrot seed and implications for industry in a changing climate
Source: PLoS One. 2017 Jun 30;12(6):e0180215. doi: 10.1371/journal.pone.0180215 (PMC5493370; doi:10.1371/journal.pone.0180215)
Supplement: S4 Table — The intercept condition is nectar glucose (μg) per ½ umbel. (DOCX) [file pone.0180215.s007.docx]

**S4 Table. Coefficients table of LM for nectar glucose:fructose ratio.** The intercept condition is nectar glucose (µg) per ½ umbel.

|  | Estimate | SE | t value | P value |
| --- | --- | --- | --- | --- |
| intercept | 58.365 | 21.387 | 2.729 | 0.007 ** |
| Fructose (µg) | 1.102 | 0.009 | 114.906 | < 0.001 *** |

Significance codes: * < 0.05, ** <0.01 *** <0.001
