# Supplementary material for: Possible mechanisms of pollination failure in hybrid carrot seed and implications for industry in a changing climate
Source: PLoS One. 2017 Jun 30;12(6):e0180215. doi: 10.1371/journal.pone.0180215 (PMC5493370; doi:10.1371/journal.pone.0180215)
Supplement: S5 Table — The final model retained time-of-day, temperature at the time of pollination, plant variety, and the interaction between time-of-day and variety. The intercept condition is the excellent variety at the peak nectar emission time of 11:00am. (DOCX) [file pone.0180215.s008.docx]

**S5 Table. Coefficients table of GLMM for nectar sugar composition.** The final model retained time-of-day, temperature at the time of pollination, plant variety, and the interaction between time-of-day and variety. The intercept condition is the excellent variety at the peak nectar emission time of 11:00am.

|  | Estimate | SE | t value | P value |
| --- | --- | --- | --- | --- |
| intercept | 1.237 | 1.469 | 0.842 | 0.400 |
| Time (04:00) | 1.371 | 1.107 | 1.238 | 0.216 |
| Time (08:00) | 0.246 | 1.085 | 0.226 | 0.821 |
| Time (14:00) | -0.331 | 0.991 | -0.334 | 0.739 |
| Time (17:00) | 0.491 | 0.994 | 0.494 | 0.622 |
| Time (20:00) | 1.312 | 1.039 | 1.263 | 0.206 |
| Time (23:00) | 0.826 | 1.095 | 0.755 | 0.450 |
| Temperature | 0.152 | 0.049 | 3.091 | 0.002 ** |
| Variety (medium) | 1.673 | 1.018 | 1.644 | 0.100 |
| Variety (poor) | -0.224 | 1.037 | -0.216 | 0.829 |
| Time (04:00) : Variety (medium) | -2.782 | 1.427 | -1.950 | 0.051 . |
| Time (08:00) : Variety (medium) | -2.426 | 1.395 | -1.739 | 0.082 . |
| Time (14:00) : Variety (medium) | -2.178 | 1.409 | -1.546 | 0.122 |
| Time (17:00) : Variety (medium) | -1.623 | 1.423 | -1.141 | 0.254 |
| Time (20:00) : Variety (medium) | -1.843 | 1.423 | -1.296 | 0.195 |
| Time (23:00) : Variety (medium) | -1.383 | 1.379 | -1.003 | 0.316 |
| Time (04:00) : Variety (poor) | -0.478 | 1.438 | -0.332 | 0.740 |
| Time (08:00) : Variety (poor) | 0.105 | 1.441 | 0.073 | 0.942 |
| Time (14:00) : Variety (poor) | -1.113 | 1.404 | -0.793 | 0.428 |
| Time (17:00) : Variety (poor) | -0.610 | 1.415 | -0.431 | 0.667 |
| Time (20:00) : Variety (poor) | -2.301 | 1.412 | -1.629 | 0.103 |
| Time (23:00) : Variety (poor) | 0.552 | 1.407 | 0.393 | 0.695 |

Significance codes: * < 0.05, ** <0.01 *** <0.001
