# Supplementary material for: Possible mechanisms of pollination failure in hybrid carrot seed and implications for industry in a changing climate
Source: PLoS One. 2017 Jun 30;12(6):e0180215. doi: 10.1371/journal.pone.0180215 (PMC5493370; doi:10.1371/journal.pone.0180215)
Supplement: S6 Table — The final model retained plant variety, temperature at the time of pollination, and time-of-day. (DOCX) [file pone.0180215.s009.docx]

**S6 Table. Coefficients table of ADONIS for nectar phenolic bouquet.** The final model retained plant variety, temperature at the time of pollination, and time-of-day.

|  | Df | Sum of Sqs | F value | P value |
| --- | --- | --- | --- | --- |
| Variety | 2 | 6.695 | 3.393 | 0.011 * |
| Temperature | 1 | 2.531 | 2.566 | 0.091 . |
| Time-of-day | 6 | 6.814 | 1.151 | 0.294 |
| Residual | 226 | 222.960 |  |  |

Significance codes: * < 0.05, ** <0.01 *** <0.001
