# Supplementary material for: Possible mechanisms of pollination failure in hybrid carrot seed and implications for industry in a changing climate
Source: PLoS One. 2017 Jun 30;12(6):e0180215. doi: 10.1371/journal.pone.0180215 (PMC5493370; doi:10.1371/journal.pone.0180215)
Supplement: S8 Table — (DOCX) [file pone.0180215.s011.docx]

**S8 Table. Coefficients table of ADONIS for floral volatiles; temperature trial.**

|  | Df | Sum of Sqs | F value | P value |
| --- | --- | --- | --- | --- |
| Temperature | 1 | 0.441 | 3.128 | 0.013 * |
| Residual | 16 | 2.256 |  |  |

**Significance codes: * < 0.05, ** <0.01 *** <0.001**
