# Supplementary material for: Possible mechanisms of pollination failure in hybrid carrot seed and implications for industry in a changing climate
Source: PLoS One. 2017 Jun 30;12(6):e0180215. doi: 10.1371/journal.pone.0180215 (PMC5493370; doi:10.1371/journal.pone.0180215)
Supplement: S1 Text — (DOCX) [file pone.0180215.s013.docx]

**S1 Text. Preliminary carrot pollen viability sampling method and results**

Carrot pollen was sampled for viability from a hybrid carrot field located near Waipara, Canterbury, New Zealand (43.055° S, 172.761° E) on 10 and 11 January 2014. Pollen samples were collected at 8 am, 11 am 2 pm, 5 pm 8 pm and 10 pm over two days. Six pollen dehiscing umbels were sampled at each time (except for 8 pm and 10 pm on 10 January where three umbels were sampled). From each umbel, three umbellets were sampled per umbel. They were selected from the outermost whorl, the innermost and a whorl equidistant from the outermost and innermost. On collection, florets were removed from each umbellet using forceps and placed directly into a cryotube that was sealed and placed immediately into into a dewar containing liquid N and transported to the laboratory for longer term storage in a -80°C freezer. Samples were assessed within three months for pollen viability. Methods for assessing the pollen viability are described in the methods section of this paper. We assessed the percentage pollen viability of 200 pollen grains per umbel. The mean (±S. E.) pollen viability across all 66 samples was 9.1 ±1.4%.
